# Spatial representativeness matters for Climate-Driven Dengue Forecasting

**DOI:** 10.1371/journal.pntd.0014270

**Published:** 2026-04-27

**Authors:** Khemmanant Khamthong, Kunwithree Phramrung

**Affiliations:** 1 Department of Mathematics, Faculty of Science, Mahasarakham University, Maha Sarakham, Thailand; 2 Mathematics and Statistics Program, Faculty of Science and Technology, Kanchanaburi Rajabhat University, Kanchanaburi, Thailand; Colorado State University, UNITED STATES OF AMERICA

## Abstract

Accurate forecasting of dengue incidence requires statistical models that explicitly accommodate overdispersion, temporal dependence, and delayed environmental forcing. We develop a Bayesian negative binomial dynamic regression model to generate monthly forecasts of dengue hemorrhagic fever (DHF) incidence in Kanchanaburi Province, Thailand. Transmission persistence is captured through lagged dengue incidence, while delayed climatic effects are represented using locally observed maximum temperature and relative humidity. Model adequacy and predictive performance are assessed using posterior predictive checks and leave-one-out cross-validation (LOO-CV). The negative binomial specification consistently outperforms Poisson-based alternatives under substantial overdispersion. Importantly, forecasting performance is not determined solely by the strength of marginal climate-dengue associations. Instead, it depends critically on the spatial representativeness of climatic inputs relative to the population at risk. Models informed by climatically representative observations yield more stable and robust out-of-sample forecasts, even when marginal associations are comparatively weaker. These findings underscore the distinction between explanatory association and predictive utility in climate-driven infectious disease models and provide practical guidance for the development of climate-informed dengue early warning systems in endemic settings.

## Author summary

Dengue fever is a mosquito-borne disease that affects hundreds of millions of people each year, particularly in tropical regions. Because dengue transmission is strongly influenced by weather conditions such as temperature and humidity, climate data are widely used to build forecasting models that help public health authorities anticipate outbreaks. However, an important practical question is often overlooked: does the location of the meteorological station used to represent climate conditions affect the reliability of dengue forecasts? In this study, we compared dengue forecasting models that used climate data from two meteorological stations located in different environments within the same province in Thailand. One station showed stronger statistical correlations with dengue incidence, while the other was located closer to the main population center where most dengue cases occur. Using Bayesian predictive model comparison, we found that the station located nearer to the population center produced forecasting performance comparable to that of the station with the stronger marginal climate-dengue correlation. These findings suggest that strong historical correlations between climate and disease do not necessarily lead to better forecasts. Instead, the spatial representativeness of climate measurements relative to the population at risk may play a key role. Our results provide practical guidance for designing climate-informed dengue early warning systems in endemic regions.

## 1. Introduction

Dengue remains a major cause of febrile illness and hospitalization across endemic regions of Southeast Asia, placing sustained pressure on local health systems and routine infectious disease surveillance [1,2]. In Thailand, recurrent dengue outbreaks continue to challenge timely preparedness, particularly at the provincial level, where surveillance decisions must often be made under uncertainty and resource constraints. Improving the ability to anticipate short-term fluctuations in dengue incidence is therefore not only a statistical exercise but also a core component of infection control planning and early warning system design.

Climate-sensitive infectious diseases such as dengue are commonly modeled using environmental covariates, motivated by well-established effects of temperature and humidity on mosquito survival, viral replication, and transmission efficiency [3,4]. However, for surveillance and operational forecasting, the utility of climate information depends not only on biological relevance but also on how well climatic measurements represent the conditions experienced by the population at risk. In practice, meteorological monitoring networks are often spatially heterogeneous, leading to a misalignment between climate data and the catchment areas of disease surveillance systems. This mismatch raises a critical question for infectious disease forecasting: whether stronger climate-dengue associations necessarily translate into better predictive performance for public health decision-making [5].

Routine dengue surveillance data present additional challenges for forecasting in endemic settings. Monthly case counts are typically overdispersed due to clustered transmission, unobserved heterogeneity, and variability in reporting practices [6]. At the same time, dengue incidence exhibits strong temporal dependence, reflecting transmission persistence and delayed responses to environmental forcing [7]. Forecasting models that fail to accommodate these features often yield unstable predictions, limiting their usefulness for infection control and early warning purposes.

Dynamic regression models with lagged incidence terms provide a pragmatic framework for capturing short-term transmission persistence while incorporating exogenous drivers such as climatic covariates [8,9]. When coupled with a negative binomial likelihood, such models can accommodate the extra-Poisson variability characteristic of infectious disease surveillance data, thereby improving predictive stability. Within a Bayesian framework, uncertainty can be coherently propagated from model parameters to forecasts, a critical requirement for risk-based decision-making in infectious disease control [10].

Many climate-dengue studies focus on identifying statistically significant associations between environmental variables and disease incidence [4,11]. However, from the perspective of infection surveillance, explanatory association does not necessarily imply predictive utility. In particular, climatic variables that exhibit strong marginal correlations with dengue incidence may offer limited forecasting benefit if they are spatially misrepresentative of exposure conditions relevant to the surveillance population. Despite its operational relevance, the role of spatial representativeness of climate inputs in shaping dengue forecast performance has received limited explicit attention in the infectious disease literature.

In this study, we develop a Bayesian negative binomial dynamic regression model to forecast monthly Dengue Hemorrhagic Fever (DHF) incidence in Kanchanaburi Province, Western Thailand. The model explicitly captures short-term transmission persistence through lagged dengue incidence and incorporates lagged climatic covariates derived from provincial meteorological stations. Rather than seeking to maximize climate-dengue association strength, the primary objective is to evaluate how the spatial representativeness of climatic inputs influences out-of-sample forecasting performance. By contrasting models informed by meteorological observations with differing degrees of alignment to the population under surveillance, we distinguish explanatory association from predictive value in a manner directly relevant to dengue surveillance and infection control.

Building on the concept of spatial exposure misalignment, we hypothesize that climate observations obtained from meteorological stations that are more spatially representative of the population under surveillance will provide equal or improved out-of-sample predictive performance compared with stations that exhibit stronger marginal climate-dengue correlations but are geographically less representative.

To test this hypothesis, we compare models informed by climate data from two meteorological stations in Kanchanaburi Province. The first station is located near the provincial population center, where most dengue cases are reported, while the second station is situated in the mountainous Thong Pha Phum district, which exhibits distinct microclimatic conditions. This comparison allows us to Evaluate whether spatial representativeness of climate inputs improves forecasting reliability for dengue surveillance.

## 2. Methods

### 2.1. Study design and data

A retrospective ecological time-series study was conducted to investigate the association between climatic variability and dengue incidence in Kanchanaburi Province, Western Thailand. Monthly dengue case counts were obtained from routine provincial surveillance records maintained by public health authorities. Meteorological variables, including monthly mean maximum temperature and monthly mean relative humidity, were retrieved from official meteorological monitoring conducted at two stations located within the province. The primary outcome was the monthly number of reported dengue cases. All datasets were aggregated at the monthly level and temporally aligned. Calendar years originally recorded in the Buddhist Era were converted to the Gregorian calendar to ensure temporal consistency across data sources and facilitate integration with meteorological records.

We evaluated two meteorological stations with contrasting geographical profiles. The Kanchanaburi station is located in the provincial administrative center, where the majority of the population resides and where dengue surveillance activities are concentrated. Climatic conditions recorded at this station are therefore expected to better represent the environmental conditions experienced by much of the population at risk.

In contrast, the Thong Pha Phum station is situated in a remote mountainous district characterized by forested terrain, lower population density, and distinct microclimatic conditions compared with the provincial population center.

This contrast provides a natural framework for evaluating the role of spatial representativeness in climate-disease modeling. Specifically, it allows us to examine whether meteorological data that are more closely aligned with the environmental conditions experienced by the surveillance population yield more reliable predictive inputs for dengue forecasting than data from stations located in climatically distinct areas, even when the latter exhibit stronger marginal correlations with dengue incidence.

### 2.2. Descriptive characteristics and exploratory analysis

To provide an initial overview of dengue transmission dynamics in the study area, we conducted an exploratory analysis of monthly dengue incidence in Kanchanaburi Province. Descriptive inspection of the time series facilitates assessment of interannual variability, outbreak clustering, and long-term temporal patterns, thereby motivating the subsequent dynamic modeling strategy.

**Fig 1:**
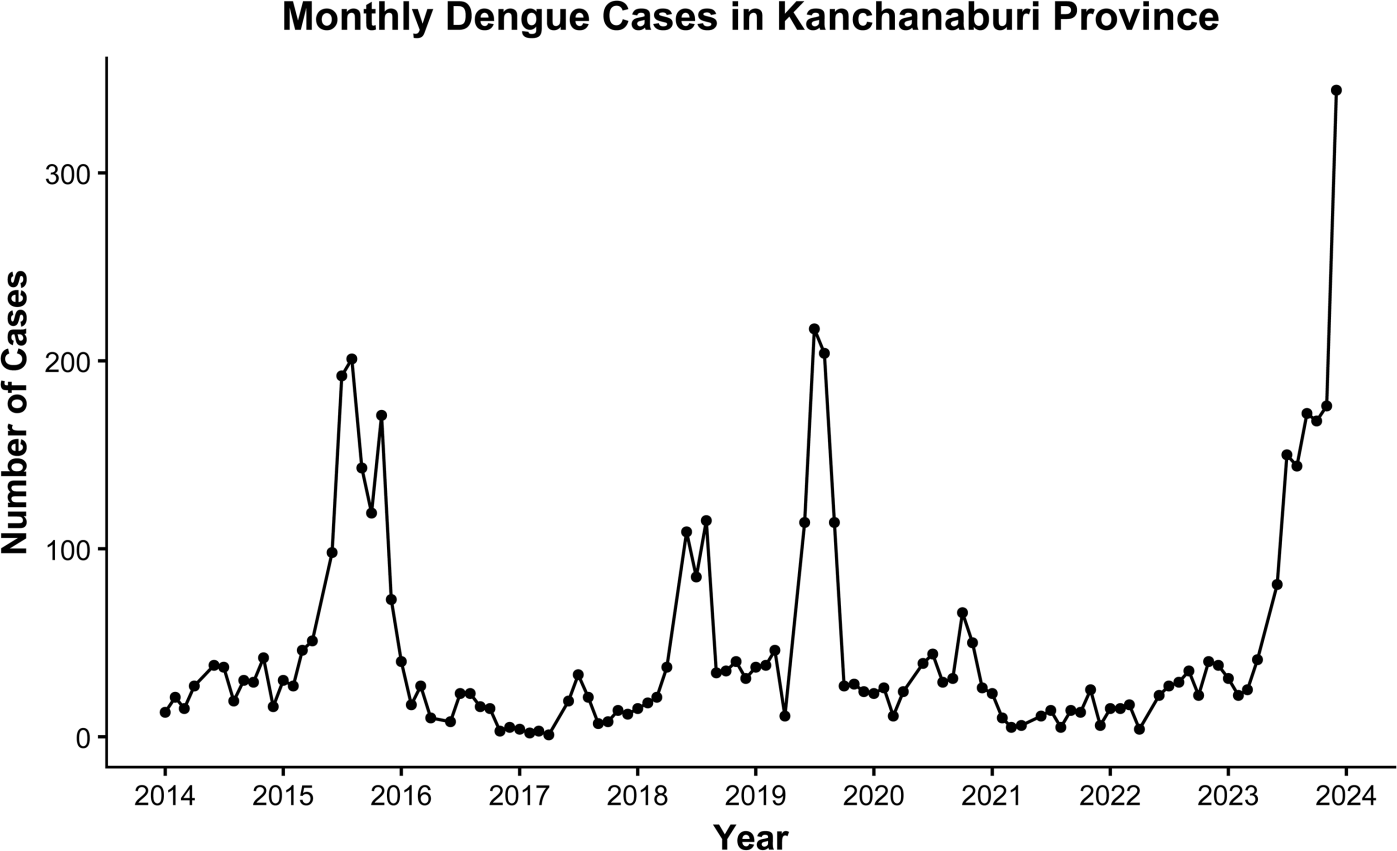
Monthly dengue cases in Kanchanaburi Province, Thailand, from 2014 to 2023. The time series displays substantial temporal variability and recurrent outbreak peaks, supporting the application of dynamic time-series modeling approaches.

Fig 1 illustrates pronounced interannual variability with recurrent outbreak peaks, indicating substantial temporal dependence and nonstationary behavior. These characteristics are consistent with dengue transmission dynamics and motivate the use of time-series regression models that explicitly account for lagged incidence and climatic drivers.

To further characterize the statistical properties of the observed data and inform model specification, Table 1 summarizes the distributional characteristics of dengue incidence and meteorological variables. Monthly dengue cases exhibit pronounced overdispersion (Mean = 46.44; SD = 56.40), providing a strong empirical rationale for adopting a negative binomial likelihood over a Poisson formulation. Furthermore, climatic variables demonstrate substantial inter-station variability, particularly in rainfall and relative humidity; for instance, the Thong Pha Phum station recorded a markedly higher mean rainfall (132.87 mm) compared to the Kanchanaburi station (88.88 mm). This reflects significant spatial heterogeneity in local microclimatic conditions, necessitating the standardization of predictors prior to model estimation.

**Table 1:** Summary statistics for monthly dengue incidence and climatic variables in Kanchanaburi Province.

Variable	Mean	SD	Q1	Median	Q3	Min	Max
*Dengue incidence*							
Monthly dengue cases (count)	46.44	56.40	15.00	27.00	42.25	1	344
*Meteorological variables: Kanchanaburi station*							
Rainfall (mm)	88.88	90.70	13.53	59.70	139.10	0.00	469.70
Maximum temperature (°C)	34.62	2.15	33.17	34.45	35.83	30.50	41.00
Relative humidity (%)	69.13	6.39	64.00	69.00	75.00	55.00	84.00
*Meteorological variables: Thong Pha Phum station*							
Rainfall (mm)	132.87	133.79	10.45	117.55	206.45	0.00	573.80
Maximum temperature (°C)	34.14	2.35	32.48	33.40	35.68	29.80	40.60
Relative humidity (%)	76.70	8.97	70.00	79.00	85.00	56.00	90.00

*Notes*: Q1 and Q3 denote the 25th and 75th percentiles, respectively. All statistics were calculated from the raw monthly dataset prior to lag construction. The dataset comprised 120 monthly observations covering the study period. The dengue surveillance dataset contained continuous observations throughout the study period, with a minimum of one reported case per month and no zero-case months (0%), indicating the absence of zero-inflation in the primary outcome variable. Meteorological variables were obtained from two official monitoring stations located in Kanchanaburi Province.

Synthesizing these observations, the temporal persistence, pronounced overdispersion, and climatic variability suggest that dengue incidence is driven by a combination of intrinsic transmission momentum and lagged environmental forcing. We therefore hypothesize that a model integrating lagged incidence with locally representative, multi-month lagged climatic covariates will more effectively capture underlying transmission dynamics and enhance out-of-sample predictive performance.

Spearman’s rank correlation coefficients (Table 2) reveal that climatic variables at the Thong Pha Phum station exhibit consistently stronger monotonic associations with dengue incidence compared to the Kanchanaburi station. Furthermore, the strong intercorrelation between rainfall and relative humidity at both sites highlights the risk of multicollinearity in frequentist regression frameworks. This underscores the advantage of our Bayesian hierarchical approach, which inherently accommodates correlated predictors and ensures coherent uncertainty propagation during inference.

**Table 2:** Spearman’s rank correlation coefficients between monthly dengue cases and climatic variables from two meteorological stations in Kanchanaburi Province.

Station	Variable	Cases	Rainfall	Max temperature	Relative humidity
Kanchanaburi	Cases	1.00	0.13	−0.06	0.18
	Rainfall	0.13	1.00	−0.03	0.75
	Max temperature	−0.06	−0.03	1.00	−0.46
	Relative humidity	0.18	0.75	−0.46	1.00
Thong Pha Phum	Cases	1.00	0.21	−0.23	0.30
	Rainfall	0.21	1.00	−0.44	0.79
	Max temperature	−0.23	−0.44	1.00	−0.80
	Relative humidity	0.30	0.79	−0.80	1.00

*Notes*: Values represent contemporaneous Spearman’s rank correlation coefficients based on complete monthly observations. Correlations are reported for exploratory purposes and do not account for lagged or nonlinear effects.

### 2.3. Feature construction and lag specification

To account for short-term temporal dependence in dengue transmission, we incorporated lagged incidence terms as predictors, a strategy commonly employed in infectious disease time-series modeling to represent intrinsic transmission persistence and reporting-related autocorrelation [6,9]. Monthly case counts were log-transformed ($\log(Y_{t}+1)$) to linearize autoregressive effects and stabilize variance. Lagged dengue incidence at one and two months was included to capture short-term transmission momentum, delayed case reporting, and unobserved local transmission dynamics commonly observed in dengue surveillance systems [6,12].

Climatic predictors consisted of monthly mean maximum temperature and mean relative humidity. Climatic variability may influence dengue transmission through cumulative effects on the life cycle of *Aedes* mosquitoes, including larval development, adult mosquito abundance, and the extrinsic incubation period of the virus, together with the subsequent time required for human infection, case detection, and reporting within surveillance systems [13,14].

To identify an appropriate lag structure, an exploratory cross-correlation analysis was conducted between climatic variables and monthly dengue incidence. The analysis indicated relatively stronger and more stable associations for maximum temperature and relative humidity at a 5-month lag compared with other candidate lags. Considering both ecological plausibility and the empirical patterns observed in the data, climatic predictors were therefore lagged by five months in the primary model specification. This lag is consistent with the cumulative ecological processes linking climate conditions to dengue transmission, including mosquito population development, viral incubation within the vector, and delays associated with human infection and surveillance reporting.

All continuous predictors were standardized (zero mean, unit variance) prior to model fitting. Consequently, estimated regression coefficients represent the expected change in dengue incidence associated with a one-standard-deviation increase in each predictor, facilitating comparison of effect sizes and improving numerical stability during Bayesian estimation [10].

### 2.4. Model specification

Let $Y_{t}$ denote the observed number of dengue cases in month *t*. Dengue incidence was modeled using a negative binomial distribution *t*o accommodate overdispersion commonly observed in infectious disease surveillance data:


$$Y_{t}\sim \text{Negative Binomial}(\mu_{t},\phi ),$$ (1)

where $\mu_{t}$ denotes the conditional mean incidence and ϕ is an overdispersion parameter governing the variance structure. Under this parameterization, the variance of $Y_{t}$ increases with $\mu_{t}$, allowing the model to capture variability exceeding the Poisson assumption.

The conditional mean was linked to covariates through a log-linear dynamic regression structure:


$$\log(\mu_{t})=\beta_{0}+\beta_{1}{\tilde{Y}}_{t-1}+\beta_{2}{\tilde{Y}}_{t-2}+\beta_{3}{\tilde{T}}_{t-5}+\beta_{4}{\tilde{H}}_{t-5},$$ (2)

where ${\tilde{Y}}_{t-1}$ and ${\tilde{Y}}_{t-2}$ denote standardized log-transformed dengue incidence lagged by one and two months, respectively; ${\tilde{T}}_{t-5}$ denotes standardized maximum temperature lagged by five months; and ${\tilde{H}}_{t-5}$ denotes standardized relative humidity lagged by five months.

This specification explicitly captures temporal dependence through lagged outcome terms rather than through stochastic autoregressive error components, thereby avoiding potential overparameterization or double-counting of serial correlation. The resulting framework aligns with established INGARCH-type modeling approaches for infectious disease time series while remaining parsimonious and interpretable at a monthly temporal scale [6,9].

## 3. Bayesian inference

### 3.1. Negative binomial formulation

Monthly dengue case counts often exhibit variability exceeding that expected under a Poisson assumption due to unobserved heterogeneity, reporting noise, and clustered transmission events. To explicitly accommodate this excess variation, dengue incidence was modeled using a negative binomial distribution parameterized by its mean $\mu_{t}$ and overdispersion parameter ϕ.

Under this parameterization, the probability mass function is given by


$$P(Y_{t}=y_{t}|\mu_{t},\phi )=\frac{\Gamma (y_{t}+\phi )}{\Gamma (\phi )\;y_{t}!}{\left(\frac{\phi }{\phi +\mu_{t}}\right)}^{\phi }{\left(\frac{\mu_{t}}{\phi +\mu_{t}}\right)}^{y_{t}},$$ (3)

where Γ(·) denotes the gamma function. The variance of $Y_{t}$ is


$$\text{Var}(Y_{t})=\mu_{t}+\frac{\mu_{t}^{2}}{\phi },$$ (4)

which exceeds the mean whenever ϕ is finite. Smaller values of ϕ correspond to greater overdispersion, while the model converges to a Poisson distribution as ϕ→∞.

This formulation allows the model to flexibly capture observed variability in dengue incidence without imposing restrictive distributional assumptions, thereby improving both in-sample fit and out-of-sample predictive performance.

Taking the logarithm of the likelihood yields the log-likelihood contribution for observation *t*:


$$\ell_{t}=\log\Gamma (Y_{t}+\phi )-\log\Gamma (\phi )-\log(Y_{t}!)+\phi \log\left(\frac{\phi }{\phi +\mu_{t}}\right)+Y_{t}\log\left(\frac{\mu_{t}}{\phi +\mu_{t}}\right).$$ (5)

The full log-likelihood is obtained by summing $\ell_{t}$ over all time points. The conditional mean $\mu_{t}$ is linked to lagged dengue incidence and climatic covariates through a log-linear dynamic regression structure, enabling explicit modeling of short-term transmission persistence and delayed environmental effects.

Bayesian inference provides a principled probabilistic framework for synthesizing observed data with prior knowledge. The posterior distribution of the parameter vector θ, given the observed dengue case series $𝐘=(Y_{1},\ldots ,Y_{T})$, is determined by Bayes’ theorem:


p(θ∣𝐘)∝p(𝐘∣θ)p(θ), (6)

where p(𝐘∣θ) represents the likelihood function and p(θ) denotes the joint prior distribution.

### 3.2. Prior specification

Weakly informative priors were specified to regularize parameter estimation while preserving data-driven inference:


$$\beta_{1},\beta_{2}\sim 𝒩(0,0.3^{2})$$ (7)

$$\beta_{3},\beta_{4}\sim 𝒩(0,0.5^{2})$$ (8)

$$\beta_{0}\sim 𝒩(3,1^{2})$$ (9)

ϕ~Exponential(1). (10)

Relatively tighter priors were assigned to lagged dengue incidence terms to reflect stronger prior expectations of short-term persistence in transmission dynamics. Slightly more diffuse priors were used for climatic covariates to allow flexibility in estimating delayed environmental effects whose magnitude and direction may vary across ecological and climatic settings.

The exponential prior on the overdispersion parameter ϕ enforces positivity and stabilizes posterior estimation while remaining weakly informative. Together, these prior choices ensure identifiability of the dynamic regression structure, mitigate overfitting, and allow posterior inference to be driven primarily by the information contained in the observed data.

### 3.3. Model assessment

Bayesian inference was carried out using Hamiltonian Monte Carlo implemented in Stan via the brms package. Four parallel chains were fitted, each run for 12,000 iterations, with the initial 4,000 iterations discarded as burn-in. The remaining draws were used for posterior inference. Chain behavior was examined to ensure stable sampling. Trace plots showed no evidence of poor mixing, effective sample sizes were adequate across parameters, and all $\hat{R}$ values were below 1.01.

Model adequacy was evaluated by comparing observed dengue counts with data replicated from the fitted models. These posterior predictive checks focused on whether the models could reproduce the overall distribution of cases and the main temporal patterns observed in the time series. Replicated data generated from the posterior predictive distribution were compared with observed case counts to assess both the distribution of cases and the temporal structure, including seasonal patterns.

Out-of-sample predictive performance was evaluated using leave-one-out cross-validation based on Pareto-smoothed importance sampling. Models were compared using expected log predictive density, with differences in ELPD and their associated standard errors used to summarize relative predictive performance. A small number of influential observations were identified based on elevated Pareto shape parameters. These observations were examined, and moment matching was applied where necessary to improve the stability of cross-validation estimates. Sensitivity to the assumed count distribution was assessed by fitting a Poisson model with the same linear predictor and comparing its predictive performance with that of the negative binomial specification. Model comparison focused on out-of-sample predictive accuracy rather than in-sample fit.

### 3.4. Sensitivity and robustness analyses

To ensure the identifiability of the dynamic regression structure and mitigate potential overfitting, we employed weakly informative priors tailored to each parameter class (Eqs. 7–10). Furthermore, we conducted extensive sensitivity analyses to verify the robustness of our primary inferences across alternative lag configurations and prior specifications. Specifically, we evaluated climatic lags ranging from four to six months. While models with shorter lags remained statistically significant, the five-month lag was prioritized for its superior alignment with the biological latency of dengue transmission. Conversely, longer lags (e.g., six months) resulted in non-significant associations for key variables, such as relative humidity, where the 95% credible interval encompassed zero. These results confirm that our core findings are not driven by arbitrary modeling assumptions but reflect stable underlying associations.

## 4. Results and discussion

In this section, we present the empirical findings from our Bayesian negative binomial framework, evaluating the drivers of dengue in Kanchanaburi Province. The analysis begins with model convergence diagnostics, followed by an assessment of transmission persistence, climatic forcing, and predictive robustness.

Table 3 presents the posterior summaries and convergence diagnostics for the Bayesian negative binomial models. All parameters demonstrated excellent mixing and convergence, as evidenced by $\hat{R}$ values of 1.00 and substantial effective sample sizes for both the bulk and tail of the posterior distributions. These metrics, alongside the results from the sensitivity analyses (3.4), confirm that our posterior inferences are robust and the Hamiltonian Monte Carlo sampling was highly efficient. Lagged dengue incidence at one and two months is positively associated with current incidence across both station-based models, reflecting the strong temporal persistence of dengue transmission. In contrast, maximum temperature and relative humidity at a five-month lag display negative associations with current dengue incidence. These effects are consistent across stations and remain robust after accounting for overdispersion and temporal dependence. The estimated dispersion parameter ϕ further confirms substantial extra-Poisson variability in monthly dengue incidence.

**Table 3:** Posterior summaries and convergence diagnostics of the Bayesian negative binomial model.

Parameter	Estimate	SE	2.5%	97.5%	$\hat{R}$	Bulk ESS	Tail ESS
*Kanchaburi Station*							
$\beta_{0}$	3.497	0.057	3.385	3.610	1.00	28,837	21,877
$\beta_{1}$	0.607	0.080	0.450	0.761	1.00	19,614	21,355
$\beta_{2}$	0.274	0.083	0.111	0.437	1.00	19,077	20,950
$\beta_{3}$	−0.335	0.068	−0.469	−0.203	1.00	22,714	21,209
$\beta_{4}$	−0.282	0.070	−0.421	−0.145	1.00	22,089	21,559
ϕ	3.371	0.519	2.457	4.478	1.00	26,151	20,586
*Thong Pha Phum Station*							
$\beta_{0}$	3.513	0.059	3.398	3.631	1.00	23,180	19,604
$\beta_{1}$	0.609	0.081	0.450	0.766	1.00	19,254	20,890
$\beta_{2}$	0.235	0.083	0.072	0.398	1.00	18,441	20,419
$\beta_{3}$	−0.443	0.105	−0.650	−0.236	1.00	16,671	19,718
$\beta_{4}$	−0.428	0.108	−0.643	−0.216	1.00	17,115	19,656
ϕ	3.077	0.465	2.253	4.067	1.00	22,827	19,637

*Notes*: Posterior estimates are reported on the log scale. $\hat{R}$ values close to 1.00 indicate convergence. Bulk ESS and Tail ESS denote effective sample sizes for the bulk and tail of the posterior distributions, respectively. All covariates were standardized prior to model fitting.

Incidence rate ratios (IRRs) derived from the posterior distributions are reported in Table 4. All covariate effects exhibit high posterior probabilities of direction, narrow credible intervals, and excellent convergence diagnostics, supporting the reliability of the inferred associations.

**Table 4:** Incidence Rate Ratios (IRR) and posterior diagnostics from the Bayesian negative binomial model for dengue incidence in Kanchanaburi.

Parameter	IRR	2.5%	97.5%	pd (%)	$\hat{R}$	ESS
*Kanchaburi Station*						
$\beta_{0}$	33.0009	29.5193	36.9615	100.00	1.000	28,782
$\beta_{1}$	1.8346	1.5679	2.1410	100.00	1.000	19,576
$\beta_{2}$	1.3158	1.1179	1.5484	99.94	1.000	19,030
$\beta_{3}$	0.7155	0.6258	0.8163	100.00	1.000	22,686
$\beta_{4}$	0.7540	0.6565	0.8651	99.98	1.000	22,026
*Thong Pha Phum Station*						
$\beta_{0}$	33.5386	29.9117	37.7619	100%	1.000	23,094
$\beta_{1}$	1.8398	1.5697	2.1509	100%	1.000	19,211
$\beta_{2}$	1.2651	1.0752	1.4894	99.77%	1.000	18,419
$\beta_{3}$	0.6428	0.5221	0.7895	100.00%	1.000	16,952
$\beta_{4}$	0.6522	0.5258	0.8057	100.00%	1.000	17,082

*Notes*: IRR were obtained by exponentiating posterior regression coefficients. Credible intervals correspond to 95% posterior intervals. The probability of direction (pd) represents the posterior probability that the effect is strictly positive or negative. $\hat{R}$ values close to 1.00 and large effective sample sizes (ESS) indicate excellent convergence and sampling efficiency. All continuous covariates were standardized prior to model fitting.

### 4.1. Model adequacy and posterior predictive diagnostics

**Fig 2:**
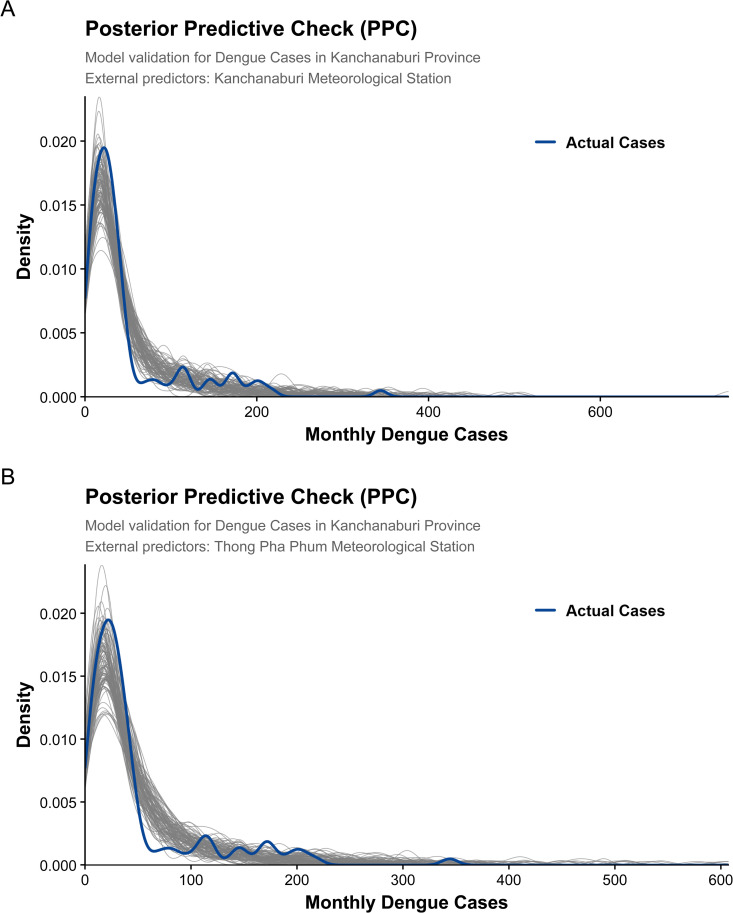
Posterior predictive check (PPC) density overlays for monthly dengue incidence. Panels (A) and (B) correspond to the two study areas. The observed dengue cases are compared with 100 replicated datasets drawn from the posterior predictive distribution of the Bayesian negative binomial model. The close agreement between observed and replicated densities indicates that the model adequately captures key marginal distributional features, including overdispersion and right-skewness.

Model adequacy was evaluated using a comprehensive set of posterior predictive checks targeting both marginal distributional fit and temporal dependence across the full study period of 120 months. Density-based posterior predictive checks (Fig 2) show close agreement between the observed dengue incidence and replicated datasets drawn from the posterior predictive distribution, indicating that the negative binomial specification adequately captures the pronounced right-skewness and overdispersion characteristic of the surveillance data.

**Fig 3:**
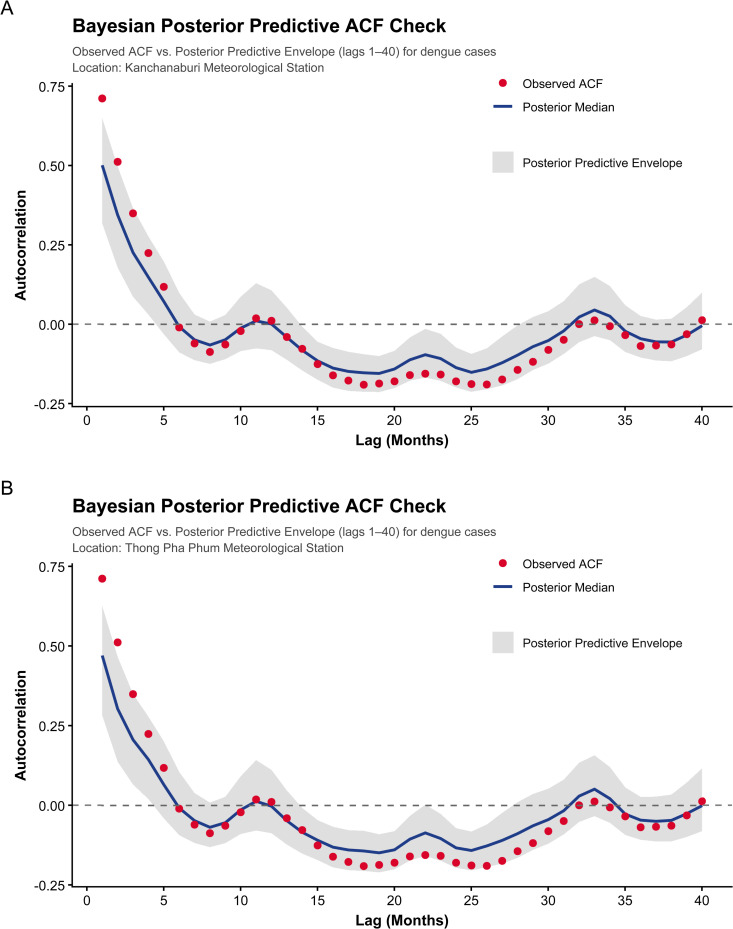
Bayesian posterior predictive autocorrelation function (ACF) checks for monthly dengue incidence. Panels (A) and (B) correspond to the two study areas. The solid blue line represents the posterior median ACF computed from replicated datasets drawn from the posterior predictive distribution; the shaded band denotes the 90% posterior predictive envelope, and the red points indicate the observed ACF. Autocorrelations are displayed up to lag 40 months to assess whether the fitted model reproduces the short- to medium-term temporal dependence observed in the data.

To assess whether the fitted model reproduces the temporal dependence observed in the data, Bayesian posterior predictive autocorrelation function (ACF) checks were conducted (Fig 3). The observed ACF (red dots) lies largely within the 90% posterior predictive envelope (gray shaded region) across lags up to 40 months. This result suggests that the inclusion of lagged dengue incidence effectively captures the dominant short-term serial dependence and the seasonal structure of the time series without requiring an additional stochastic autoregressive error component.

**Fig 4:**
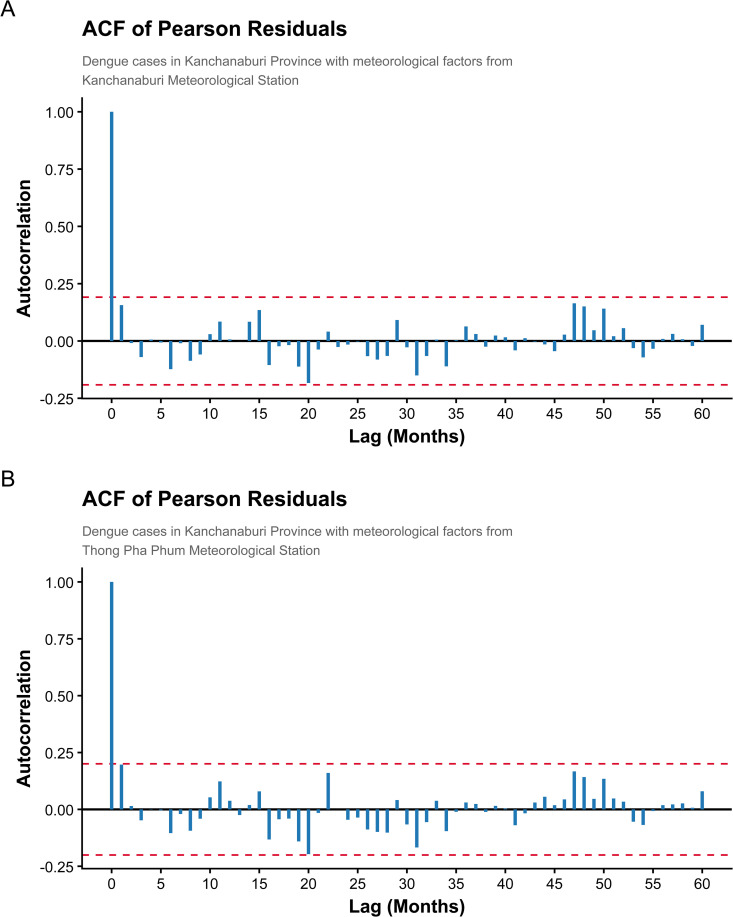
Autocorrelation function (ACF) of Bayesian-consistent Pearson residuals from the negative binomial regression model with lagged dengue cases and climate covariates. Panels (A) and (B) correspond to the two study areas. The dashed lines denote approximate 95% confidence bounds under the white-noise assumption. Residual autocorrelation is examined up to 60-month lags to evaluate potential long-term temporal structure, including the documented 3-5 year dengue outbreak cycles. The absence of substantial residual autocorrelation across lags indicates that temporal dependence is adequately captured by the lagged response structure without the need for an additional stochastic autoregressive error component.

To further evaluate longer-term temporal structure, residual diagnostics based on Bayesian-consistent Pearson residuals were examined up to 60-month lags (Fig 4). This extended lag window allows assessment of potential interannual dependence associated with the 3–5-year dengue outbreak cycles reported in Southeast Asia. Across both study areas, residual autocorrelations remain within the nominal 95% confidence bounds, indicating no substantial unexplained long-term temporal dependence.

Taken together, these posterior predictive and residual diagnostics suggest that the fitted model provides an adequate representation of both the distributional properties and the temporal dynamics of monthly dengue incidence, capturing short-term transmission persistence as well as longer-term interannual variability.

Table 5 presents the model comparison results based on leave-one-out cross-validation (LOO). Across all specifications, the Bayesian negative binomial model consistently demonstrates improved predictive performance relative to the Poisson alternative, as evidenced by higher expected log predictive density (ELPD) and lower LOOIC values. This underscores the importance of explicitly accounting for overdispersion in monthly dengue incidence data.

**Table 5:** Model comparison based on leave-one-out cross-validation (LOO) and predictive accuracy.

Model	Station	LOOIC	RMSE	ELPD	ΔELPD	SE
Negative Binomial	Kanchanaburi	901.48	32.77	−450.74	0.00	0.00
Negative Binomial	Thong Pha Phum	912.44	33.49	−456.22	−5.48	2.60
Poisson	Kanchanaburi	1911.84	30.02	−955.92	−505.18	144.41
Poisson	Thong Pha Phum	2022.73	31.02	−1011.36	−560.62	161.06

Differences in ΔELPD exceed twice their associated standard errors, indicating that model ranking is statistically meaningful rather than driven by sampling variability.

Regarding the comparison between meteorological sources, the negative binomial model informed by the Kanchanaburi station data yields the lowest nominal LOOIC and RMSE. However, its predictive performance is statistically indistinguishable from the model utilizing Thong Pha Phum data. Specifically, the difference in expected log predictive density (ΔELPD =−5.48) is smaller than twice its associated standard error (SE = 2.60), indicating that the two station-based models are statistically equivalent in terms of predictive accuracy based on the available data.

These findings suggest that stronger marginal correlations between climatic variables and dengue incidence do not necessarily translate into superior out-of-sample predictive performance. Although climatic variables from the Thong Pha Phum station exhibit stronger marginal associations with dengue cases, the resulting model does not demonstrate a statistically meaningful improvement in predictive accuracy.

Taken together, these results support the interpretation that spatial representativeness of climatic exposure relative to the primary population center may influence predictive performance but does not necessarily confer a statistically significant predictive advantage. In this context, meteorological observations that more closely reflect the environmental conditions experienced by the majority of the population can provide predictive information comparable to that derived from stations located in climatically distinct areas, even when the latter exhibit stronger marginal correlations with dengue incidence.

### 4.2. Out-of-sample forecasting performance

**Fig 5:**
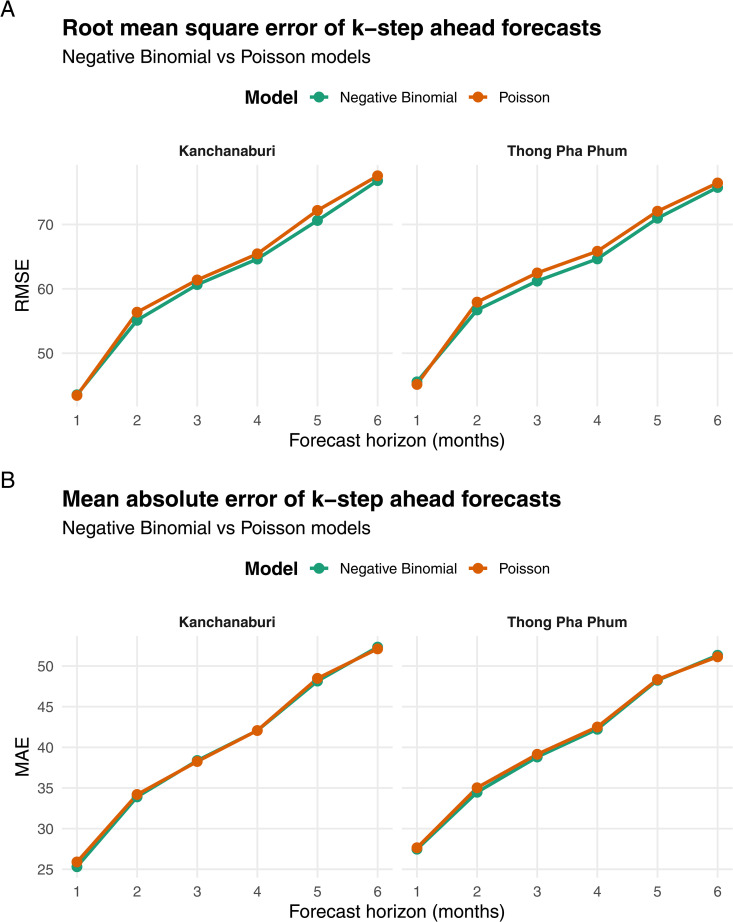
Out-of-sample forecasting performance across *k*-step-ahead horizons (1-6 months). Panels (A) and (B) report RMSE and MAE, respectively, comparing the predictive accuracy of Bayesian negative binomial (teal) and Poisson (orange) models. Climate covariates from the Kanchanaburi and Thong Pha Phum meteorological stations are used to assess model stability and spatial representativeness. Both metrics indicate that the negative binomial model consistently outperforms the Poisson model across all forecast horizons and study areas.

Out-of-sample forecasting performance was evaluated using a direct multi-step forecasting protocol with horizons ranging from one to six months ahead. Fig 5 summarizes forecasting accuracy in terms of RMSE and MAE.

Across all forecast horizons, the Bayesian negative binomial model consistently outperforms the Poisson specification for both meteorological stations. The performance advantage of the negative binomial model becomes more pronounced at longer horizons (k≥4), indicating greater robustness to overdispersion and increasing forecast uncertainty. Forecasting errors obtained from models incorporating climatic covariates from the Kanchanaburi station are broadly comparable to those derived from the Thong Pha Phum station. Although the Kanchanaburi-based models occasionally exhibit slightly lower RMSE and MAE at longer forecast horizons, the magnitude of these differences remains small. The pattern of forecasting accuracy across horizons therefore aligns with the LOO cross-validation results, which indicate that the predictive performances of the two station-based models are statistically equivalent.

The comparison further indicates that stronger marginal associations between climatic variables and dengue incidence, observed for the Thong Pha Phum station, do not necessarily translate into improved out-of-sample forecasting accuracy.

### 4.3. Discussion

This study examined whether the spatial alignment between meteorological observations and the population under surveillance influences the accuracy of climate-driven dengue forecasting. The results indicate that stronger marginal climate-dengue associations do not necessarily translate into superior out-of-sample predictive performance. Although climatic variables from the Thong Pha Phum station exhibited higher statistical correlations with dengue incidence, the predictive performances of the two station-based models were statistically equivalent. Specifically, the difference in ELPD was within two standard errors, indicating that the Kanchanaburi station-despite its lower marginal correlation-provided a predictive signal of comparable quality. This finding underscores an important distinction in infectious disease modeling: strong explanatory associations in historical data do not necessarily guarantee improved predictive utility.

#### 4.3.1. Potential mechanisms of spatial representativeness.

The observed statistical equivalence between a highly correlated distal station and a moderately correlated representative station may be explained by the mechanisms of environmental exposure. Dengue transmission is a localized process driven by the abundance and infection dynamics of *Aedes* mosquitoes in areas where human-vector contact occurs. The Kanchanaburi station, situated in the provincial administrative center, captures climatic conditions experienced by the majority of the population. In urbanized environments, microclimatic factors such as urban heat island effects, dense housing structures, and localized water storage practices can create breeding conditions that differ from those in forested or mountainous districts such as Thong Pha Phum. Consequently, meteorological observations from geographically distant environments may reflect broader regional climatic variability without accurately representing the microclimatic drivers of transmission in the primary population center.

#### 4.3.2. Generalizability and regional implications.

The implications of these findings extend beyond the present study area. The influence of spatial representativeness is likely to become more pronounced in geographically large or environmentally heterogeneous regions where climatic gradients vary across relatively short distances. For example, in a large metropolitan area such as Bangkok, meteorological measurements from a single central monitoring station may not capture the diverse microclimates that characterize densely populated urban neighborhoods and suburban districts. Conversely, in environmentally homogeneous regions, the choice of meteorological station may have a smaller influence on predictive performance. These considerations suggest that the geographical correspondence between meteorological monitoring networks and disease surveillance catchment areas represents an important structural factor in climate-informed infectious disease forecasting.

#### 4.3.3. Practical framework for dengue early warning systems.

To translate these findings into operational guidance, a systematic workflow for selecting climatic inputs in dengue early warning systems can be proposed. First, public health agencies should map the spatial distribution of the population at risk and identify the primary exposure zones within the surveillance catchment area. Second, meteorological monitoring locations should be evaluated in relation to these exposure zones, with priority given to stations located within or near densely populated areas. Third, candidate climatic predictors should be assessed using predictive validation metrics, such as leave-one-out cross-validation or out-of-sample forecasting error, rather than relying solely on historical correlation coefficients. Finally, forecasting models should explicitly account for temporal dependence and overdispersion in surveillance data to ensure realistic uncertainty estimates and robust predictive performance.

Taken together, these findings highlight an important principle for climate-informed infectious disease forecasting. The strength of historical climate-disease associations alone is not a reliable indicator of predictive utility. Instead, the spatial representativeness of environmental measurements relative to the population under surveillance appears to be a key determinant of predictive reliability.

By explicitly distinguishing explanatory correlation from predictive performance, this study provides an empirical framework for integrating meteorological observations into dengue early warning systems. Incorporating spatially representative climate inputs, together with models that account for temporal dependence and overdispersion in surveillance data, may improve the robustness of operational dengue forecasting in endemic regions.

#### 4.3.4. Study limitations.

Despite these insights, several limitations warrant consideration. First, our analysis relied on monthly aggregated surveillance data, which may overlook finer temporal fluctuations and reporting delays occurring at weekly or daily scales. Second, as the study focused on two stations within a single province, the observed spatial representativeness effects might vary in regions with different topographies or denser monitoring networks. Third, while maximum temperature and relative humidity are key drivers of mosquito ecology, other factors, such as rainfall patterns, land-use changes, and vector control interventions, also influence local transmission but were not explicitly captured in our model.

Future research integrating higher-resolution environmental data and multi-province surveillance systems will be essential to further clarify how spatial exposure misalignment shapes climate-driven forecasting across diverse ecological contexts.

## 5. Conclusion

This study analyzed monthly dengue incidence in Kanchanaburi Province using a Bayesian negative binomial time-series framework that accounted for temporal dependence, overdispersion, and climatic covariates. The observed dengue counts exhibited substantial extra-Poisson variability and strong serial correlation, features that were not adequately captured by Poisson-based formulations and motivated the use of a negative binomial specification.

Lagged dengue incidence accounted for a large proportion of short-term variation in reported cases, indicating persistent transmission dynamics and unobserved local influences. After controlling for temporal dependence, lagged maximum temperature and relative humidity remained associated with dengue incidence. These associations were consistent across meteorological stations and were supported by posterior predictive diagnostics, indicating stable model estimation.

Model comparison based on leave-one-out cross-validation showed higher out-of-sample predictive accuracy for Bayesian negative binomial models relative to Poisson alternatives. Differences in predictive performance across station-specific climate inputs were small relative to posterior uncertainty, indicating that stronger marginal climate-dengue associations were not systematically associated with improved forecasts. This result suggests that correlation strength alone provides limited guidance for selecting climatic inputs in predictive models.

Assessment of multi-step-ahead forecasts showed that the negative binomial framework maintained comparable predictive performance across lead times of up to six months. Predictive behavior at longer horizons reflected explicit representation of temporal dependence and uncertainty propagation rather than reliance on short-term associations.

Overall, the findings indicate that dengue forecasting performance is influenced more by the combination of dynamic transmission structure and the spatial representativeness of climatic inputs than by the magnitude of marginal climate-dengue associations. Bayesian negative binomial time-series models provide a suitable framework for dengue surveillance and forecasting in settings characterized by overdispersed incidence data and spatially heterogeneous climate information. Extensions incorporating spatially resolved climate data, vector surveillance indicators, or mechanistic transmission components may be considered to examine their effects on predictive behavior.

### 5.1. Ethics Approval and Consent to Participate

This study analyzed aggregated and anonymized secondary dengue surveillance data and did not involve individual-level human subjects. Ethical approval and informed consent were therefore not required in accordance with national regulations and institutional guidelines.

## References

[1] World Health Organization. Dengue: Guidelines for diagnosis, treatment, prevention and control. Geneva: WHO Press. 2009. 23762963

[2] Gubler DJ. Dengue and dengue hemorrhagic fever. Clin Microbiol Rev. 1998;11(3):480–96. doi: 10.1128/CMR.11.3.480 9665979 PMC88892

[3] Lambrechts L, Paaijmans KP, Fansiri T, Carrington LB, Kramer LD, Thomas MB, et al. Impact of daily temperature fluctuations on dengue virus transmission by Aedes aegypti. Proc Natl Acad Sci U S A. 2011;108(18):7460–5. doi: 10.1073/pnas.1101377108 21502510 PMC3088608

[4] Morin CW, Comrie AC, Ernst K. Climate and dengue transmission: evidence and implications. Environ Health Perspect. 2013;121(11–12):1264–72. doi: 10.1289/ehp.1306556 24058050 PMC3855512

[5] Naish S, Dale P, Mackenzie JS, McBride J, Mengersen K, Tong S. Climate change and dengue: a critical and systematic review of quantitative modelling approaches. BMC Infect Dis. 2014;14:167. doi: 10.1186/1471-2334-14-167 24669859 PMC3986908

[6] Held L, Höhle M, Hofmann M. A statistical framework for the analysis of multivariate infectious disease surveillance counts. Stat Model. 2005;5(3):187–99. doi: 10.1191/1471082X05st098oa

[7] Xu L, Stige LC, Chan K-S, Zhou J, Yang J, Sang S, et al. Climate variation drives dengue dynamics. Proc Natl Acad Sci U S A. 2017;114(1):113–8. doi: 10.1073/pnas.1618558114 27940911 PMC5224358

[8] Held L, Meyer S, Bracher J. Probabilistic forecasting in infectious disease epidemiology. Biom J. 2017;59(2):242–58. doi: 10.1002/bimj.201600003 28656694

[9] Meyer S, Held L, Höhle M. Spatio-temporal analysis of epidemic phenomena using the R package surveillance. J Stat Softw. 2017;77(11):1–55. doi: 10.18637/jss.v077.i11

[10] Gelman A. Scaling regression inputs by dividing by two standard deviations. Stat Med. 2008;27(15):2865–73. doi: 10.1002/sim.3107 17960576

[11] Liu Z, Zhang Z, Lai Z. Temperature variability and dengue fever transmission in China: A multi-city study. Environ Res. 2020;182:109115. doi: 10.1016/j.envres.2019.10911531923850

[12] Held L, Höhle M, Hofmann M. A statistical framework for the analysis of multivariate infectious disease surveillance counts. Biostatistics. 2006;7(4):531–48. doi: 10.1093/biostatistics/kxj020

[13] Hii YL, Rocklöv J, Ng N, Tang CS, Pang FY, Sauerborn R. Climate variability and increase in intensity and magnitude of dengue incidence in Singapore. Glob Health Action. 2009;2:10.3402/gha.v2i0.2036. doi: 10.3402/gha.v2i0.2036 20052380 PMC2799326

[14] Lowe R, Gasparrini A, Van Meerbeeck CJ, Lippi CA, Mahon R, Trotman AR, et al. Nonlinear and delayed impacts of climate on dengue risk in Barbados: A modelling study. PLoS Med. 2018;15(7):e1002613. doi: 10.1371/journal.pmed.1002613 30016319 PMC6049902

